# Life satisfaction among elderly patients in Nepal: associations with nutritional and mental well-being

**DOI:** 10.1186/s12955-018-0947-2

**Published:** 2018-06-07

**Authors:** Saruna Ghimire, Binaya Kumar Baral, Isha Karmacharya, Karen Callahan, Shiva Raj Mishra

**Affiliations:** 1 Agrata Health and Education (AHEAD)-Nepal, Kathmandu, Nepal; 2 Department of Biochemistry, Nepal Medical College and Teaching Hospital, Kathmandu, Nepal; 3 School of Health and Allied Sciences, Pokhara University, Lekhnath, Nepal; 4 School of Community Health Sciences, University of Nevada Las Vegas (UNLV), 4505 S. Maryland Parkway, Las Vegas, USA; 5 Nepal Development Society, Bharatpur-10, Nepal

**Keywords:** MNA, Nepal, Elderly, Nutritional assessment, Depression, GDS, Life satisfaction, Mediation, Moderation

## Abstract

**Background:**

Nepal’s demography is aging rapidly, yet few studies to date have examined how this has affected the health and well-being of the elderly, defined as those above 60 years in Nepal’s Senior Citizen Act (2006). Our study, abbreviated NepEldQOLII, aims to assess perceived life satisfaction, and evaluate its relationship with nutritional health and mental well-being among the burgeoning Nepalese elderly population.

**Methods:**

A cross-sectional survey among 289 Nepalese elderly, aged ≥60 years, attending an outpatient clinic of a hospital in Kathmandu, Nepal was conducted. Nutritional status, depression, and life satisfaction were assessed by a mini-nutritional assessment scale (range: 0–14), a geriatric depression scale (range: 0–15), and a satisfaction with life scale (range: 5–35), respectively. Mediation analyses, adjusted for age, sex, marital status, and family type, were used to assess mediating relationships between nutritional and mental wellbeing with life satisfaction as the outcome.

**Results:**

Approximately 21% of the participants were dissatisfied with their life. Life satisfaction was positively associated with being married, high family income, involvement in active earning, and a high nutritional score. Conversely, life satisfaction was inversely associated with living in a nuclear (as opposed to joint) family, the perception of having worse health than peers, the perception of being ignored/hated due to old age, and a higher depression score. In mediation analyses, both nutrition (β = 0.48, bias-corrected and accelerated (BCa) 95% CI: 0.27, 0.69) and depression (β = − 0.87, BCa 95% CI: -1.01, − 0.74) had significant direct associations with life satisfaction. Furthermore, both nutrition (β = 0.30, BCa 95% CI: 0.13, 0.49) and depression (β = − 0.07, BCa 95% CI: -0.14, − 0.03) mediate each other’s association with life satisfaction. Nutritional score mediated 7% of the total association between depression and life satisfaction; depression mediated 38% of the total association between nutrition and life satisfaction.

**Conclusions:**

Life satisfaction shows a pattern of decline as nutritional and mental health status decrease. Both depression and under-nutrition had a significant association with life satisfaction. The pathway by which nutrition affects life satisfaction is influenced by depression as a mediator. Moreover, nutritional status explained a small portion of the relationship between depression and life satisfaction. These observed preliminary findings should be confirmed in future longitudinal studies.

## Background

Nepal, like many other countries in South Asia, has been successful in lowering mortality rates [1] as well as increasing life expectancy from 62.5 years in 2000 to 69.2 years in 2015 [2]. Consequently, the population of elderly, defined as adults 60 years of age and above by the Nepali Senior Citizens Act [3], has increased from 1.5 million in 2001 to 2.2 million in 2011 [4]. This represents a 3.5% growth rate for the elderly and exceeds the 1.4% overall population growth rate in Nepal [4]. This substantial growth mandates urgent preparation in Nepal to address the specific social, psychological, economic, and health needs of the elderly. Yet, comprehensive studies on health, nutrition, and quality of life, including both health-related quality of life (HRQOL) as well as life satisfaction, among Nepalese elderly are lacking.

Life satisfaction, a general measure of overall wellbeing [5], measures the degree of coherence between the life dreamed of and the life achieved [6]. More specifically, life satisfaction, reflecting more of the psychological dimension, focuses on the “feeling” component or subjective well-being [5]. In previous studies, life satisfaction has been associated with positive health behaviors [7], better physical and mental health outcomes [8], and longevity [8]. Thus, life satisfaction can serve as a general indicator of health risk [7] and successful aging [9].

In recognition of its importance, research evaluating life satisfaction among the elderly has increased globally [7, 10, 11], but such research is limited in Nepal. The only study from Nepal, conducted among Nepalese elderly who lived with their son, found that financial satisfaction, education, functional status, self-perceived health, and instrumental support from the son were the strongest correlates of life satisfaction [12]. Given that life satisfaction is positively related to individuals’ social support [7] and inversely related to solitude [11], and that in the Nepalese society, sons are traditionally the caretakers of their parents and obliged to provide them with financial and social support, it follows logically that older adults with a son are more likely to be satisfied with their life; thus, the findings of the study [12] may not be generalizable to Nepalese elderly not living with a son or without a family. Additionally, inferences about life satisfaction among Nepalese elderly cannot be reliably made from studies conducted in other countries due to potentially contrasting social and cultural values [13]. Moreover, no study of Nepalese elderly has yet examined the interrelationship between life satisfaction, depression, and nutritional status.

Malnutrition and depression, two common comorbidities in older adults [14–16], show a bidirectional relationship. Depression is a significant contributing factor to weight loss and malnutrition among the elderly [14, 15]. Conversely, adequate nutritional status is associated with lower odds of depression [16, 17]. Moreover, both malnutrition and depression influence life satisfaction in older adults [10, 18]. Therefore, based on evidence gleaned so far, the relationship between malnutrition and depression and life satisfaction is rather complex and cannot fully be explained by traditional multivariate methods. Mediational analysis, which allows for the simultaneous evaluation of predictors as mediators and moderators or the estimation of the extent to which mediators contribute to the observed association between an exposure and an outcome, is one way that a researcher can explain the process or mechanism by which one variable affects another [19]. Despite the plausibility of some mediating and moderating relationship between life satisfaction, nutrition, and depression, to the best of our knowledge, no study has yet explored the mediation-moderation relationship among the trio in any population. Therefore, we also aimed to explore the mediating relationships between life satisfaction, nutrition, and depression.

**Fig. 1:**
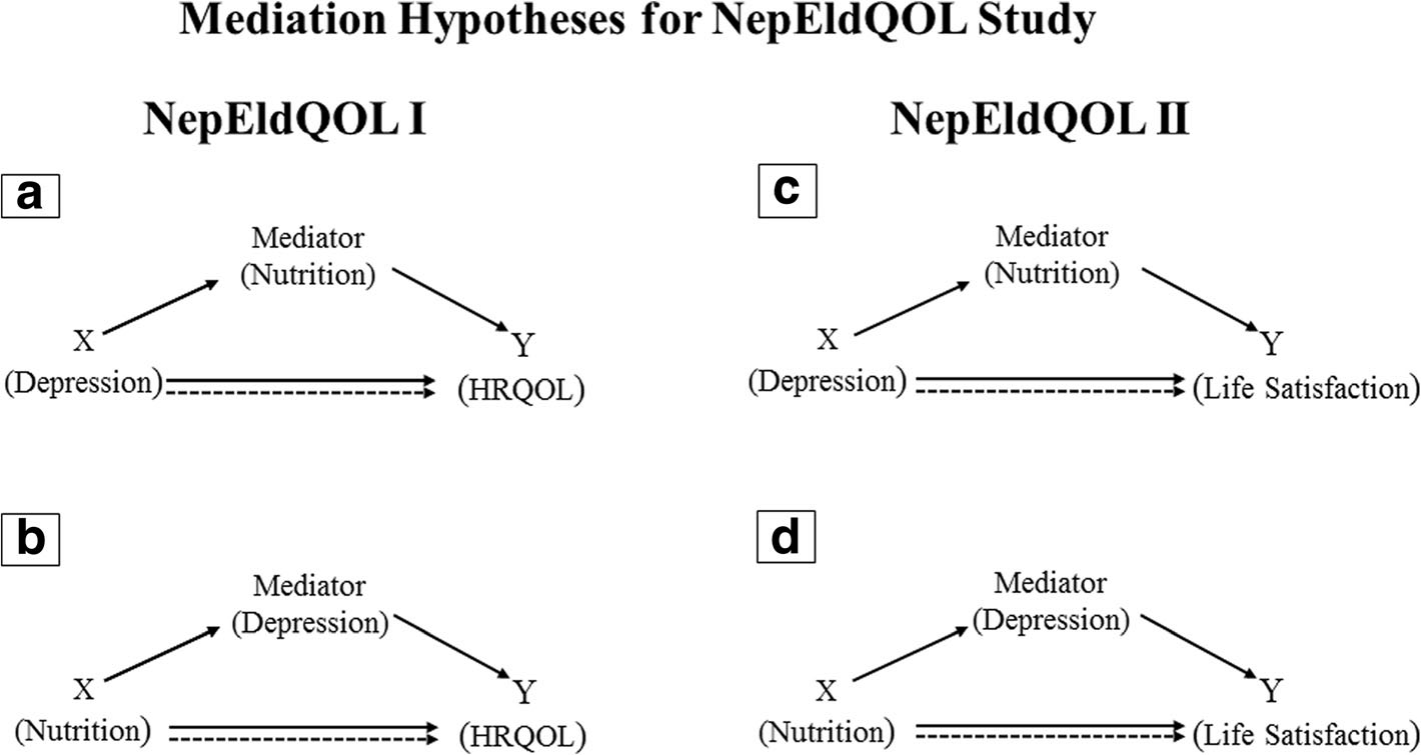
Mediation model for the association between **a** depression and health-related quality of life (HRQOL), mediated by nutrition in NepEldQOL I; **b** nutrition and HRQOL, mediated by depression in NepEldQOL I; **c** depression and life satisfaction, mediated by nutrition in NepEldQOL II; **d** nutrition and life satisfaction, mediated by depression in NepEldQOL II. X: predictor variable; Y: outcome variable

In our previous quality of life study, the NepEldQOL I, we explored the potential role of nutritional wellbeing in explaining the association between depression and HRQOL, using mediation analyses (Fig. 1). Following the same procedures, this current study, NepEldQOL II, explores depression as a potential mediator of the relationship between nutrition and life-satisfaction, a measure of subjective well-being that complements the HRQOL (Fig. 1). By assessing both health-related quality of life and life satisfaction, the combined NepEldQOL I and II studies provide the most comprehensive portrayal of the well-being of Nepal’s elderly population to date.

Therefore, the aims of the NepEldQOL II study are twofold. First, we aim to assess life satisfaction among a sample of Nepalese elderly and evaluate the correlates of life satisfaction. Second, we aim to evaluate the mediating role of nutritional status and depression in the life satisfaction-nutrition-depression triad. We hypothesize that while depression plays a direct role in overall life satisfaction among Nepalese elderly, some of the relationship can be explained by the mediating role of nutritional status; likewise, we hypothesize a mediating role for depression in explaining the relationship between nutritional status and life satisfaction.

## Methods

### Study setting

The current study, NepEldQOL II, is the second of a two-part study which aimed to evaluate the overall quality of life of Nepalese elderly. The NepEldQOL I research is fully described elsewhere (Ghimire et al, Health-related quality of life among Nepalese elderly, forthcoming). Briefly, a cross-sectional study was conducted from January–April 2017 in the Out-Patient Department (OPD) of the Nepal Medical College and Teaching Hospital in Kathmandu, Nepal.

### Study procedure

The sample size for this cross-sectional study was calculated in StatCalc in Epi Info 7, (sample size = z^2^pq/d^2^). Selecting 0.05 as an acceptable error rate (alpha) and 95% confidence intervals, and using the 24% prevalence of malnutrition among Nepalese elderly found in another study [20], we obtained a sample size of 289. Criteria for inclusion in the study included being at least age 60, attending clinic on one of the survey days, and consenting to participate. Those too frail physically or mentally to respond, and/or with hearing or speech impairment were excluded. Participants were selected by systematic random sampling; from the OPD patient list, every third patient attending clinic on one of the survey days was approached for eligibility and consent. If the approached patient was deemed ineligible, then the next eligible respondent from the OPD list was approached. Written and/or verbal consent was obtained from participants, depending upon the literacy status of the subject; there were no refusals. Data were collected until required sample size was reached.

### Data collection and variables

Data were collected during patients’ wait time using individual face-to-face interviews conducted by trained graduate students in medicine and public health.

Participants’ life satisfaction was measured with the widely-used Satisfaction with Life Scale (SWLS) [20]. SWLS has good convergent validity with other types of assessments of subjective well-being [21]. Cronbach’s alpha, a measure of scale reliability, for the SWLS in this study was 0.93. In short, the SWLS is a 5-item scale; response for each item is captured by a 7-point Likert scale of agreement. The cumulative SWLS score ranges from 5 to 35 with higher scores indicating higher levels of life satisfaction. Cumulative SWLS scores are dichotomized into two categories: satisfied (SWLS> 20) and dissatisfied (SWLS≤20) [20].

Nutritional status was assessed using the Mini Nutritional Assessment – Short Form (MNA-SF) [22], a tool previously validated in multiple settings [22], including Nepal [23]. The Cronbach’s alpha for the MNA-SF scale was 0.59. A detailed description of the MNA-SF tool is provided elsewhere [22]. Briefly, the cumulative scores of the six MNA-SF items (ranging from 0 to 14) are classified into three groups: malnourished (MNA-SF score < 8), at risk of malnutrition (MNA-SF score of 8–11), and normal nutritional status (MNA-SF ≥ 12) [22].

Depression was measured using the short form of the Geriatric Depression Scale (GDS) [24], another tool which has been validated in Nepal specifically [25], as well as many different settings [26]. The GDS has been extensively described elsewhere [24]. Briefly, it is a 15-item instrument; each item has a dichotomized response in “Yes/No” format. The cumulative score of the GDS ranges from 0 to 15: 0–4 suggests no depression; 5–9 suggests mild depression; 10–15 suggests severe depression [24, 27]. In this study, the Cronbach’s alpha for the GDS scale was 0.81.

Height and weight were measured using a mechanical stadiometer and a digital weighing scale, respectively. Subsequently, body mass index (BMI) was calculated. Participant’s weight status was characterized as underweight (BMI < 18.5) normal weight (18.5 ≤ BMI < 23), overweight (23 ≤ BMI < 27.5), and obese (BMI ≥27.5) as per the World Health Organization (WHO) recommendation for Asians [28]. Socio-demographic variables assessed by self-report were age (continuous and categorized in 5-year intervals), sex, ethnicity (three groups: Upper Caste, Janjatis, or Dalit/other ethnic minority), marital status (married or separated/widow/single), family’s monthly income (continuous and categorized as < $200, $200- < $300, and ≥ $300), family structure (nuclear, joint or extended), smoking, alcohol use, and education level, which was categorized as illiterate, informal (no formal schooling but some literacy), and formal (any formal education). Also assessed by self-report were participants’ self-perceived health status (better/similar/worse) compared to peers of their age as well as perception of being ignored/hated for being old (yes/no).

### Data processing and statistical analysis

Data management and analyses were done in EpiData and IBM SPSS22 (SPSS Inc. Chicago IL, USA), respectively. Continuous variables were described with mean ± standard deviation (SD); categorical variables with frequencies. Differences in mean and frequency distributions between the groups were assessed using independent t-tests and Pearson’s chi-square (χ^2^) tests, respectively. The correlation of the SWLS score with the MNA-SF and GDS scores was given by Spearman’s correlation coefficient (ϱ). Predictors of life satisfaction were assessed in linear regression models using cumulative SWLS scores as the dependent variable. Models were adjusted for age, sex, marital status, and family type. We used bootstrap models with 5000 replications to calculate stable estimates of correlates (Tables 2, 3 and 4).

A mediational analysis [29] was conducted to determine the direct and mediating associations of depression and nutritional status on life satisfaction. Two different mediating models were developed to examine whether, and to what extent, the mediator, (nutrition in Model 1 and depression in Model 2), explained the relationship between the exposure (depression in Model 1 and nutrition in Model 2) and SWLS as the outcome. For moderation analysis, an interaction between the GDS and MNA-SF score was added to the regression model with the GDS and MNA-SF scores as predictors and SWLS as the outcome. For testing our mediation-moderation models, we used the non-parametric bootstrap approach which allows decomposition of the total effect of an exposure into natural direct and indirect effects [29]. To be consistent with the terminology used in mediation analysis [29], we use the term “effect” rather than “association” for the findings pertaining to mediational analyses. However, based on the cross-sectional nature of our study, no conclusions should be made regarding the direction of causality. The PROCESS macro for SPSS, which tests mediation models by comparing the observed indirect associations against 5000 bootstrapped resamples, was used for the mediation-moderation analyses [29]. The coefficients of associations and their bias-corrected and accelerated (BCa) 95% confidence intervals (CI) were obtained from 5000 random bootstrap samples. BCa confidence intervals that do not include zero indicate statistically significant estimates [29]. The mediation analyses were first run without any covariates (Model 1), then adjusted for age and sex (Model 2), and further adjusted for age, sex, ethnicity, marital status, educational status, smoking, alcohol use, perception of being ignored/hated, and perceived health status compared to peers (Model 3). For all statistical tests in Table 1, two-tailed *p*-values< 0.05 were considered statistically significant.

**Table 1:** Characteristics of the participants by satisfaction with life score

Characteristics	Cumulative Satisfaction with Life Score		Satisfaction with Life Score Categorized		
			Dissatisfied *n* = 61, 21.1%	Satisfied *n* = 228, 78.9%	*p*-value
	Mean ± SD	*p*-value^1^	n (%)	n (%)	
Life Satisfaction Score (Mean ± SD)	–	–	15.5 ± 3.6	27.1 ± 3.2	< 0.001^a^
MNA-SF Score (Mean ± SD)	–	–	9.2 ± 3.0	11.4 ± 2.2	< 0.001^a^
Nutritional Status		< 0.001			< 0.001
Malnourished	19.7 ± 6.3		17 (27.9)	13 (5.7)	
At risk of Malnutrition	23.4 ± 6.0		29 (47.5)	80 (35.1)	
Normal Nutritional Status	26.5 ± 4.6		15 (24.6)	135 (59.2)	
GDS Score (Mean ± SD)	–	–	9.1 ± 3.3	5.0 ± 3.4	< 0.001^a^
Depression		< 0.001			< 0.001
No	28.2 ± 4.0		7 (11.5)	116 (50.9)	
Mild	23.3 ± 5.1		23 (37.7)	80 (35.1)	
Severe	19.8 ± 5.2		31 (50.8)	32 (14.0)	
Age, Years (Mean ± SD)	–	–	69.1 ± 7.4	68.4 ± 6.2	0.435^a^
Age Categories		0.976			0.926
60–64	24.7 ± 6.3		17 (27.9)	68 (29.8)	
65–69	24.7 ± 6.4		18 (29.5)	69 (30.3)	
70–74	24.3 ± 4.7		13 (21.3)	51 (22.4)	
75 and above	24.8 ± 4.9		13 (21.3)	40 (17.5)	
Sex		0.426			0.399
Male	24.9 ± 5.7		33 (54.1)	137 (60.1)	
Female	24.3 ± 5.9		28 (45.9)	91 (39.9)	
Ethnicity		0.228			0.139
Upper Caste	25.1 ± 6.2		27 (44.3)	107 (46.9)	
Janjatis	24.4 ± 5.4		26 (42.6)	108 (47.4)	
Dalit/Other Ethnic Minorities	22.9 ± 5.3		8 (13.1)	13 (5.7)	
Marital Status		0.005			0.002
Married	25.1 ± 5.5		40 (65.6)	191 (83.8)	
Separated/Widow/Single	22.7 ± 6.4		21 (34.4)	37 (16.2)	
Educational Status		0.183			0.416
Illiterate	24.2 ± 6.2		27 (44.3)	85 (37.3)	
Informal	24.2 ± 5.1		21 (34.4)	76 (33.3)	
Formal	25.6 ± 5.8		13 (21.3)	67 (29.4)	
Family’s Monthly Income $, *n* = 167 (Mean ± SD)	–	–	174.8 ± 86.7	212.3 ± 89.7	0.036^a^
Family’s Monthly Income Category		0.109			0.101
< $200	23.9 ± 6.4		18 (58.1)	52 (38.2)	
$200- < $300	25.5 ± 4.0		7 (22.6)	55 (40.4)	
> =300	25.9 ± 5.1		6 (19.4)	29 (21.3)	
BMI (Mean ± SD)	–	–	24.4 ± 3.2	25.0 ± 3.6	0.215^a^
Weight Status		0.660			0.700
Underweight	23.0 ± 7.4		3 (4.9)	7 (3.1)	
Normal Weight	24.9 ± 6.9		18 (29.5)	59 (25.9)	
Overweight	24.4 ± 5.5		30 (49.2)	112 (49.1)	
Obese	25.1 ± 4.4		10 (16.4)	50 (21.9)	
Family Structure		0.008			0.009
Nuclear	22.4 ± 7.4		17 (27.9)	30 (13.2)	
Joint	25.2 ± 5.4		34 (55.7)	170 (74.6)	
Extended	24.1 ± 5.1		10 (16.4)	28 (12.3)	
Smoked	24.2 ± 5.6	0.147	32 (52.5)	123 (53.9)	0.102
Used Alcohol	25.1 ± 4.7	0.286	13 (21.3)	85 (37.3)	0.019
Self-Perceived Health Status		< 0.001			< 0.001
Better than Peers	25.6 ± 6.9		13 (21.3)	56 (24.6)	
Similar to Peers	25.7 ± 4.6		17 (27.9)	122 (53.5)	
Worse than Peers	21.9 ± 5.7		31 (50.8)	50 (21.9)	
Currently Working		0.017			0.067
Yes	27.1 ± 4.3		2 (3.3)	25 (11.0)	
No	24.4 ± 5.9		59 (96.7)	203 (89.0)	
Perceived as Ignored/Hated due to Old Age		< 0.001			0.001
Yes	21.1 ± 6.3		18 (29.5)	27 (11.8)	
No	25.3 ± 5.4		43 (70.5)	201 (88.2)	

*Abbreviations*: *SD* standard deviation, *BMI* body mass index, *MNA-SF* mini nutritional assessment short form cumulative score, *GDS* geriatric depression scale short form cumulative score

^a^*p*-value from independent t-test; all others are from chi-square. ^1^*p*-value from ANOVA and independent t-test as applicable.

## Results

The mean SWLS score was 24.6 ± 5.8. Approximately 21% of the participants were dissatisfied (SWLS≤20) with their life. The SWLS score was correlated positively with the MNA-SF score (ϱ =0.40, *p* < 0.001) and negatively with the GDS score (ϱ = − 0.65, *p* < 0.001). A greater proportion of participants satisfied with their life were married, had higher family income, lived in a joint family, had better-perceived health status compared to peers, were not depressed or ignored/hated due to old age, and had normal nutritional status (Table 1).

### Predictors of life satisfaction

In adjusted analyses (Table 2), married participants were significantly more satisfied with their life than those who were separated/widow/single (β = 2.19; BCa 95% CI = 0.28, 4.18). Compared to participants living in a joint family, those living in a nuclear family were significantly less satisfied (β = − 2.37; BCa 95% CI = − 4.79, 0.01). Family monthly income was positively associated with life satisfaction (β = 0.01; BCa 95% CI = 0.01, 0.02). Likewise, working participants were more satisfied with life than those retired or unemployed (β = 2.24; BCa 95% CI = 0.27, 4.17). The perception of worse health than peers (β = − 3.53; BCa 95% CI = − 4.94, − 2.08) and the perception of being ignored/hated due to old age (β = − 3.95; BCa 95% CI = − 6.07, − 1.88) were associated with a lower life satisfaction score. Life satisfaction was positively associated with the MNA-SF nutritional score (β = 0.90; BCa 95% CI = 0.65, 1.17) and inversely associated with the GDS depression score (β = − 1.00; BCa 95% CI = − 1.14, − 0.85) (Table 2).

**Table 2:** Linear regression for factors associated with life satisfaction among Nepal’s elderly

	Unadjusted		Adjusted	
	β	BCa 95% CI	β	BCa 95% CI
Age	−0.01	−0.11, 0.09	0.01	−0.09, 0.12
Sex-Female (Reference: Male)	−0.55	−1.92, 0.79	− 0.24	−1.63, 1.08
Ethnicity (Ref: Upper Caste)				
Janjatis	−0.39	− 1.72, 0.93	− 0.43	− 1.71, 0.94
Dalit/Other Ethnic Minority	−1.86	−4.34, 0.59	− 1.01	−3.47, 1.38
Marital Status- Married (Ref: Separated/Widow/Single)	**2.38**	**0.60, 4.20**	**2.19**	**0.28, 4.18**
Education (Ref: Illiterate)				
Informal	−0.59	−1.92, 0.75	−0.75	−2.06, 0.53
Formal	1.40	−0.12, 2.89	0.97	−0.84, 2.72
Family’s Monthly Income $	**0.01**	**0.01, 0.02**	**0.01**	**0.01, 0.02**
Family Structure (Ref: Joint)				
Nuclear	**−2.65**	**−4.86, − 0.44**	**− 2.37**	**−4.79, 0.01**
Extended	− 0.60	− 2.36, 1.20	− 0.89	− 2.71, 0.84
Self-Perceived Health Status (Ref: Similar to Peers)				
Better than Peers	1.31	−0.56, 3.08	1.55	−0.31, 3.34
Worse than Peers	**−3.75**	**−5.18, −2.30**	**−3.53**	**−4.94, − 2.08**
Currently Working - Yes (Reference: No)	**2.78**	**0.93, 4.42**	**2.24**	**0.27, 4.17**
Perceived as ignored/hated due to old age - Yes (Reference: No)	**−4.16**	**−6.13, −2.20**	**−3.95**	**−6.07, −1.88**
MNA-SF cumulative score	**0.92**	**0.67, 1.17**	**0.90**	**0.65, 1.17**
GDS cumulative score	**−0.99**	**−1.12, − 0.86**	**−1.00**	**− 1.14, − 0.85**

β: unstandardized coefficient, BCa: bias-corrected and accelerated, 5000 bootstrap samples

Statistically significant associations are highlighted in bold

Adjusted for age, sex, marital status, and family type

*Abbreviations*: *BMI* body mass index, *MNASF* mini nutritional assessment short form, *GDS* geriatric depression scale

### Mediation-moderation analyses

In moderation analysis, there was no statistically significant interaction between nutrition and depression (β = 0.03, BCa 95% CI: -0.02, 0.08).

### Depression as a mediator of the nutrition – Life satisfaction association

Mediation analysis with depression, as captured by the cumulative score on the GDS assessment, as the mediator, revealed a partial mediation in the relationship between nutrition and life satisfaction, through depression (Table 3). In the final model, adjusted for age, sex, ethnicity, marital status, smoking, alcohol use, educational status, the perception of being ignored/hated and perceived health status compared to peers, a significant indirect association was found in the mediation model (β = 0.30, BCa 95% CI: 0.13, 0.49). Depression mediated 38% of the total associations of nutrition on life satisfaction (Table 3).

**Table 3:** Mediation analysis for the association between nutrition and life satisfaction, mediated by depression

	Model 1		Model 2		Model 3	
	β	BCa 95% CI	β	BCa 95% CI	β	BCa 95% CI
Total effect, (c)	0.92	0.67, 1.16	0.94	0.69, 1.18	0.78	0.52, 1.03
Direct effect, (c’)	0.53	0.33, 0.74	0.56	0.36, 0.76	0.48	0.27, 0.69
Indirect effect, (a X b)	0.39	0.23, 0.57	0.37	0.21, 0.56	0.30	0.13, 0.49
^a^Ratio of indirect to total effect mediated, (a X b/c)	0.42	0.27, 0.60	0.40	0.25, 0.57	0.38	0.19, 0.60
^a^Ratio of indirect to direct effect, (a X b/c’)	0.72	0.37, 1.51	0.67	0.33, 1.33	0.62	0.24, 1.49

Model 1: unadjusted mediational model; Model 2: adjusted for age, and sex; Model 3: adjusted for age, sex, ethnicity, marital status, smoking, alcohol use, educational status, perception of ignored/hated, perceived health status compared to peers

Number of bootstrap samples for bias-corrected bootstrap confidence intervals: 5000

β: Unstandardized coefficient; BCa: Bias-corrected and accelerated

^a^Values are ratios of regression coefficients

### Nutrition as a mediator of the depression - life satisfaction association

Mediation analysis (Table 4), with the MNA-SF nutritional score as the mediator, revealed a partial mediation in the relationship between depression and life satisfaction, through the MNA-SF score. In the final model, adjusted for age, sex, ethnicity, marital status, smoking, alcohol use, educational status, the perception of being ignored/hated, and perceived health status compared to peers, a small but significant indirect association was found in the mediation model (β = − 0.07, BCa 95% CI: -0.14, − 0.03). The nutritional score mediated 7% of the total association of depression on life satisfaction (Table 4).

**Table 4:** Mediation analysis for the association between depression and life satisfaction, mediated by nutrition

	Model 1		Model 2		Model 2	
	β	BCa 95% CI	β	BCa 95% CI	β	BCa 95% CI
Total effect, (c)	−0.99	−1.13, − 0.85	−1.02	− 1.16, − 0.88	−0.94	−1.08, − 0.81
Direct effect, (c’)	−0.89	−1.02, − 0.75	−0.92	−1.05, − 0.78	−0.87	−1.01, − 0.74
Indirect effect, (a X b)	− 0.10	−0.17, − 0.05	−0.10	− 0.17, − 0.05	−0.07	− 0.14, − 0.03
^a^Ratio of indirect to total effect mediated (a X b/c)	0.11	0.05, 0.18	0.10	0.05, 0.17	0.07	0.03, 0.14
^a^Ratio of indirect to direct effect (a X b/c’)	0.12	0.06, 0.21	0.12	0.06, 0.21	0.08	0.03, 0.16

Model 1: unadjusted mediational model; Model 2: adjusted for age, and sex; Model 3: adjusted for age, sex, ethnicity, marital status, smoking, alcohol use, educational status, perception of being ignored/hated, perceived health status compared to peers

Number of bootstrap samples for bias-corrected bootstrap confidence intervals: 5000

β: Unstandardized coefficient; BCa: Bias-corrected and accelerated

^a^Values are ratios of regression coefficients

## Discussion

This is a pioneer study assessing the life satisfaction of Nepalese elderly. Consistent with a recent report suggesting that Nepalese people are overall satisfied with their life [30], our study showed four in five elderly adults were satisfied with their lives. This is a notable finding against the backdrop that Nepal ranks as the 17th poorest country globally, with less than $1000 gross domestic income per capita. The World Happiness Index ranks Nepal as the third happiest country in South Asia and 99th happiest country worldwide [30]. However, not captured by these matrices is the finding that one in five elderly people were NOT satisfied with their life. If this finding is generalizable to Nepal’s entire elderly population of 2.2 million [4], it would translate into 464,200 Nepalese elderly dissatisfied with their lives, which reflects poorly on their overall well-being.

Our findings of positive associations between life satisfaction and employment [10, 12, 31], family income [10, 12, 31], higher education level [10, 12], being married [10, 32], and living in a joint family [32, 33] are consistent with other studies. Socioeconomic status impacts life satisfaction in terms of affordability of daily needs [34], access to health care [35], and adequate social support [32]. Likewise, the health benefits conferred by marriage and living in a family can be attributed to better social, psychological, and financial support received from spouse and family members [32, 36, 37].

Life satisfaction shows a pattern of decline as nutritional health decreases, seen in this study and others [38, 39]. Optimal nutrition can decrease the risk of morbidity and mortality from many illnesses [18, 40], and can positively influence self-perceived health [40] and life satisfaction [41]. Daily consumption of fruits and vegetables have been strongly associated with increased happiness, greater life satisfaction, and well-being [41]. Notably, food intake is not just the physical act of eating food but also involves sociological and cultural aspects of eating [40]. As such, the positive psychological and social aspects associated with mealtime makes the experience more satisfying [40]. Thus, the pathway from nutrition to positive well-being and improved life satisfaction is possibly influenced more by positive meal experiences and to a lesser extent by nutrition itself [40].

Our finding of a significant inverse relationship between depression and life satisfaction is also supported by other studies [10, 42, 43]. Life dissatisfaction is more frequent in people with mental health problems than in the general population [7, 42]. Adults who are dissatisfied with life are over 41 times more likely to have depressive symptoms than those who are satisfied with life [10]. Life dissatisfaction is an effective indicator of individuals with depression, suicidal tendencies, and other psychiatric illnesses and disabilities [7, 42]. Additionally, the bidirectional relationship between malnutrition and depression [14–16] explains the fact that both depression and malnutrition could partially mediate each other’s association on life satisfaction in the current study. The link between poor nutrition and depression is biologically plausible [44, 45]; inflammation, a decrease in antioxidant levels, and oxidative and nitrosative stress [46] may explain the role of several nutrients in the mechanisms of depression. Likewise, depression can influence food choice and intake through behavioral changes like meal skipping and disordered eating [47]. Although prior studies have contributed to describing the complex relationships between nutrition, depression, and life satisfaction, to the best of our knowledge, none so far have evaluated their interrelationship in the same model. Here, we provide a preliminary explanation of this triadic relationship through our mediation-moderation analyses. While a well-defined causal pathway to higher life satisfaction remains elusive, we demonstrated here the importance of mental health and nutrition.

### Strengths and limitations

This is the first study not only to assess the life satisfaction of Nepalese elderly but also to explore the relationship of three important aspects of biological aging- depression, nutritional status, with life satisfaction - in the Nepalese context. Further, to our knowledge, this is the first study to explore the mediating role of depression and elderly malnutrition on life satisfaction among the elderly in any context. The study findings suggest that life satisfaction is truly multi-faceted [40], and depression plays a crucial role. Further prospective studies are needed to identify the direction of this relationship.

Naturally, our study is subject to some limitations. Due to the cross-sectional nature of our data, no inferences should be made with regard to the direction of causality; the possibility of reverse causation cannot be ruled out. We acknowledge that mediation models are best suited for longitudinal data. However, through this analysis, we provide preliminary support to the hypothesized mediating role of depression on life satisfaction. Future research should evaluate this hypothesis with prospective data. There may be other mediators in the pathway between nutrition and/or depression and life satisfaction which we were not able to consider in this study; future studies should explore other potentially important mediators. Our study participants were patients attending outpatient clinics in an urban setting; however, the nutritional status, depression, and life satisfaction of the general population and those in a rural setting may differ significantly from them, which would impact the generalizability of our results. Lastly, the internal consistency of the MNA-SF scale was relatively low in our study (Cronbach’s α = 0.59); however, given that the MNA-SF has already been validated among the Nepalese elderly population [23], we accept it as a valid nutritional assessment tool for this context.

## Conclusions

Life satisfaction among Nepali elderly shows a pattern of decline as nutritional and mental health decreases. Both depression and under-nutrition had a significant association with life satisfaction. The pathway by which nutrition affects life satisfaction was found to be influenced by depression. Conversely, nutritional status explained a small portion of the relationship between depression and life satisfaction. The observed preliminary findings await confirmation in future longitudinal studies.

## Abbreviations

BCa: Bias-Corrected and accelerated
BMI: Body mass index
GDS: Geriatric depression scale
MNA-SF: Mini Nutritional Assessment-Short Form
OPD: Out-patient department
QoL: Quality of life
SD: Standard deviation
SWLS: Satisfaction with life scale
